# Temporal Bacterial Surveillance of Salmon Aquaculture Sites Indicates a Long Lasting Benthic Impact With Minimal Recovery

**DOI:** 10.3389/fmicb.2018.03054

**Published:** 2018-12-12

**Authors:** Joost T. P. Verhoeven, Flora Salvo, Robyn Knight, Dounia Hamoutene, Suzanne C. Dufour

**Affiliations:** ^1^Department of Biology, Memorial University of Newfoundland, St. John’s, NL, Canada; ^2^Aquaculture, Biotechnology and Aquatic Animal Health Section, Fisheries and Oceans Canada, Northwest Atlantic Fisheries Center, St. John’s, NL, Canada

**Keywords:** flocculent matter, aquaculture microbiology, bacterial communities, biomarkers, benthic indicators, biodiversity, waste products

## Abstract

Coastal aquaculture has experienced substantial growth in the last few decades and associated impacts on natural environments are of increasing importance. Understanding both the effects of aquaculture on marine ecosystems and the processes of recovery during fallowing periods is crucial for the development of a more environmentally sustainable industry. Because bacteria are sensitive to environmental change, surveying fluctuations in bacterial communities is a promising tool for monitoring the status of benthic environments. Here, we used 16S rRNA gene high-throughput sequencing to characterize bacterial communities in flocculent matter samples collected over a period of 3 years and at various distances from cages (0–200 meters) at production and fallow (3–35 months) salmon aquaculture sites in southern Newfoundland to evaluate the environmental impact of aquaculture on predominantly hard-bottom substrates. Bacterial composition analysis revealed four clusters, three of which (defined as “recently disturbed,” “intermediate impact,” and “high impact”) differed markedly from a fourth “low impact” cluster that contained far-field samples collected >500 m from cages. Samples within the high impact group were most often collected directly under cages, whereas those in the intermediate impact group were mainly sampled from 20 to 40 m from cages. Large scale phylum shifts (increases of Bacteroidetes, Firmicutes, Spirochaetes, and decreases in Proteobacteria and Epsilonbacteraeota) and a decline in bacterial diversity were observed in the high impact cluster, indicating significant ecological change. Samples from sites of different fallow duration were found in the high impact cluster, indicating a lack of recovery, even after 35 months of fallowing. Finally, we identified 28 genera as bacterial biomarkers, specific to one or more clusters, including genera associated with organically enriched environments and previously reported in the context of aquaculture impacts. Tracking the relative abundance of biomarkers in relation to different lengths of fallowing in the three more impacted clusters showed that these markers remained significantly above low impact cluster levels at all times, further pointing toward incomplete recovery. Our results suggest that coastal aquaculture on hard-bottom substrates is prone to long lasting impacts on bacterial communities, especially below cages, and that effects can be accurately tracked using bacterial community profiles or specific biomarkers.

## Introduction

Aquaculture is a rapidly growing industry, contributing nearly 50% of the fish consumed on a global scale (FAO, 2016). In Canada, aquaculture has experienced substantial growth, with a 63% increase in production value between 2003 and 2013 (Fisheries and Oceans Canada, 2013), and salmon aquaculture is largely responsible for this increase. Canada is the third-largest salmon producer globally (Agriculture and Agri-Food Canada, 2017), and the salmon aquaculture industry is particularly important to the province of Newfoundland (NL), where it provides economic growth and employment opportunities, especially for rural communities along the South coast. In NL, salmon are cultured in deep sheltered bays that have hard-bottom substrates consisting mostly of rock and cobble with patches of natural sediment (Hamoutene et al., 2013, 2015; Hamoutene, 2014). Salmon are kept in suspended net pens over water depths greater than 30 m throughout a 1 to 2-year growth period and are fed fish pellets until harvesting (Hamoutene et al., 2013, 2015; Hamoutene, 2014), at which point a fallow period may be implemented (Fisheries and Oceans Canada, 2015).

As aquaculture expands, there is growing concern over potential impacts on the marine ecosystem. The seafloor within the vicinity of cages at salmon aquaculture sites may receive substantial inputs of particulate matter (Carroll et al., 2003; Jusup et al., 2009). This particulate matter consists mostly of decaying fish-feed pellets, fish fecal matter, microbes and other organic matter. In aquaculture impact studies (and herein) this material is referred to as flocculent matter (DFO, 2014; Salvo et al., 2015; Hamoutene et al., 2016; Verhoeven et al., 2016) due to its characteristic flocculent appearance when it deposits and remains on the benthic surface (Yokoyama et al., 2006), where it can drive significant benthic community changes (Ye et al., 1991).

Accumulation of flocculent matter causes an increase in oxygen consumption by bacteria and consequently lowers oxygen availability within the sediment, creating hypoxic and anoxic conditions along with elevated concentrations of sulfides and/or methane (Hargrave et al., 2008). Such biogeochemical changes may negatively impact naturally occurring communities, often leading to their decline (Jusup et al., 2009; Salvo et al., 2017). The effects of organic enrichment on the benthos have been widely studied, especially on soft substrates, and aquaculture activities typically lead to changes in macrofaunal succession (Karakassis, 2000) and decreases in species diversity (Zhulay et al., 2015). Benthic bacterial communities have also been shown to shift with changes in organic matter concentrations near cages (Kawahara et al., 2009; Cordier et al., 2018; Keeley et al., 2018).

The monitoring of flocculent matter deposition is routinely completed by investigating redox potential, sulfides, and/or changes in faunal communities in sediment obtained using grabs or cores (Hargrave et al., 2008). However, these methods differ in their sensitivity to detect subtle effects of organic enrichment, e.g., along a gradient of distance from cages (Carroll et al., 2003), and are difficult to implement where sites are dominated by hard substrates, such as in NL (Hamoutene et al., 2013). To overcome these obstacles, drop-camera visualization is currently being utilized in NL to monitor organic enrichment (Fisheries and Oceans Canada, 2015). Drop-camera monitoring along the south coast of NL has revealed that white microbial mats and opportunistic polychaete complexes (OPC) are indicators of organic enrichment as they are closely associated with the accumulation of organic matter at aquaculture sites (Salvo et al., 2015, 2017). However, this method has its drawbacks as it only provides a visual representation of the conditions at the seafloor and lacks the ability to clearly detect intermediate levels of disturbance (Salvo et al., 2017).

Since the degradation of organic matter is mainly driven by microorganisms, identifying the microbes present in flocculent matter may prove to be a valuable approach for investigating and monitoring organic enrichment at aquaculture sites. Bacterial communities can respond rapidly to environmental changes, including those resulting from organic loading due to aquaculture activity (Nogales et al., 2011). The composition of bacterial communities in sediments from aquaculture sites has been examined using cloning and sequencing techniques or, more recently, high-throughput sequencing. Differences in sedimentary bacterial community composition between farm and non-farm sites (Kawahara et al., 2009), and along organic enrichment gradients at fish farms (Dowle et al., 2015; Cordier et al., 2018; Keeley et al., 2018; Stoeck et al., 2018) highlight the potential for using bacterial communities as bioindicators of aquaculture impact. Interestingly, bacterial community responses were similar to those shown by macrofaunal bioindicators of aquaculture activity (Stoeck et al., 2018), supporting the concept of using bacterial indicators to assess aquaculture-linked environmental perturbations. Bacterial indicators should also be useful to track remediation processes, although studies of bacterial succession during fallowing periods are lacking. Fallow recovery time varies depending on location and can span from a few months to >5 years for a full recovery (Keeley et al., 2014).

The development of locally adapted bacterial bioindicator approaches relies on knowledge of both the composition of bacterial communities and their response to aquaculture (and fallowing) in the sites of interest. While bacterial community patterns in fish farm sediments from regions as distant as Norway, Chile, Tasmania and New Zealand can be similar (Stoeck et al., 2018), bacterial taxonomic structure may differ in flocculent matter from NL aquaculture sites. Due to the paucity of (natural) soft sediment at NL sites, flocculent matter generally settles on rocky substrates, transforming the benthic habitat; bacterial colonization and succession pathways in this flocculent layer likely differ from those occurring elsewhere, where waste settles atop soft sediments that harbor a vastly different resident microbiota.

In Newfoundland, an examination of bacterial communities in flocculent matter sampled from an aquaculture site after 3 months of fallowing revealed the presence of potential indicator taxa, including bacteria recovered from microbial mat samples (Verhoeven et al., 2016). Given the limited spatial and temporal scope of the initial study, further research is needed to adequately characterize the effects of enrichment and fallowing based recovery on benthic bacterial community structures in this region, with the goal of eventually developing reliable monitoring tools and adequate strategies for ensuring ecological sustainability. In this study, we sampled flocculent matter along transects at fallow and production sites over a 3-year period. We collected grab samples from areas with visible flocculent matter, characterized bacterial taxa via 16S rRNA gene high-throughput sequencing on isolated DNA and examined bacterial community structure and its relationship to distance to cage and fallowing duration.

## Materials and Methods

### Study Sites

The three study sites were located on the southern coast of NL, where substrates are predominantly rocky; the exact location of those sites is not disclosed at the request of the site owners. Site A, located near Hermitage Bay, was stocked in May 2016 and remained in production during both sampling events, in October 2016 (3 months production) and June 2017 (12 months production). This site was previously sampled in September 2015, 3 months into a fallowing period that lasted 11 months (Verhoeven et al., 2016). The long-term fallow site (site B), located in Belle Bay, was first stocked in 2005 and underwent multiple production cycles before the start of fallowing in August 2014. Sampling of this site took place in August 2016 (24 months into fallow) and on June 2017 (35 months into fallow). A third production site (site C, 24 months production), also located in Belle Bay, was sampled (at harvest) in June 2017.

### Flocculent Matter and Sediment Sampling

Samples were collected at each of the described study sites along predefined transects running perpendicular or parallel to the shore and cage alignment. At each station in a transect, situated 0, 20, 40, 80, 120, 160, and 200 m from cages, video footage was taken using drop camera to visualize the benthic environment. Three additional stations in the middle of four-cage groupings were investigated at the production site (site A) in October 2016.

An Ekman grab (6″ × 6″ × 10″) was used to collect samples, which were categorized as either naturally occurring sediments or aquaculture associated flocculent matter; herein, substrates from all but the far-field stations described below were qualified as flocculent matter, but we acknowledge that stations along the transects likely contained some proportion of natural sediments as well. At stations along transects where drop camera revealed either bare bedrock with no significant collectable material or boulders that could interfere with successful grab operation, sampling was not attempted. As aquaculture-associated flocculent matter can be heterogeneous on a small scale (Verhoeven et al., 2016), we collected up to three sub-samples from each successful grab (i.e., instances when the grab closed on the bottom and at least 25% its volume was filled with substrate) using a sterile polyester swab (Starplex Scientific Inc.), to better capture the existing bacterial diversity.

Collection of naturally occurring sediments not impacted by aquaculture operations was complicated by the scarcity of such natural sediment in the sampling regions; however, a small number of sediment samples from “far-field- stations” (>500 m from fallow site B) were successfully collected (*N* = 3) in June 2017 and included here to serve as “reference.” Swabs (total *N* = 119, collected from 38 stations, Supplementary Table S1) were stored in a transport medium (Starswab Multitrans System, Starplex Scientific Inc.), and kept on ice until returning to shore, where they were temporarily frozen at -20°C until returning to the laboratory where samples were subsequently stored at -80°C.

### Water Sampling

To test the potential for detecting flocculent matter-associated bacterial indicator taxa within the water column close to the substrate as an alternative to grab sampling, a small number of water samples (*N* = 4, Supplementary Table S1) were collected in 2016 at sites A and B, at 0 and 200 m from cage edge (at both sites). Roughly 10 L of seawater was collected using a Niskin bottle, carefully lowered as to not disturb the flocculent matter present and triggered while suspended ∼1 m from the seafloor. Upon returning to shore, the entire volume of each water sample was filtered sequentially through a Whatman GF/A 0.45 μM filter (Whatman plc) and a Durapore 0.22 μM filter (Millipore Sigma Inc.). Filters were then stored at -20°C until returning to the laboratory and were subsequently stored at -80°C.

### Total Organic Carbon Measurement

Flocculent matter and natural sediment samples (*N* = 41, Supplementary Table S2) used for measurement of percentage total organic carbon (%TOC) were freeze-dried and, after removing any visible shells and debris, homogenized using a mortar and pestle. Acidification was performed by the addition of 50 μL 1.2 N hydrochloric acid and incubating until dry for up to 2 h. The acidification was repeated four times to ensure the removal of inorganic carbon. Samples were loaded into tin capsules and %TOC was measured using a CE-440 Elemental Analyzer (Exeter Analytical, Coventry, United Kingdom) with Acetanilide as internal standard. Differences in %TOC were assessed using a multiple-comparison one-way ANOVA using the Tukey multiple comparisons test (GraphPad Prism v7, GraphPad Software Inc.).

### Nucleic Acid Extraction

Flocculent matter, natural sediment, and water filter samples were thawed on ice, after which flocculent matter and natural sediment samples were vortexed thoroughly for 30 s to create a homogeneous mixture. Two-hundred microliters of flocculent matter or natural sediment mixture, and 1 cm^2^ of sterile excised 0.22 μM filter paper was used for nucleic acid isolation with the AllPrep PowerViral DNA/RNA Kit (Qiagen) according to manufacturer instructions.

### 16S rRNA Gene Sequencing and Processing

Bacterial 16S rRNA gene sequencing was outsourced to the Integrated Microbiome Resource of the Centre for Comparative Genomics and Evolutionary Bioinformatics (Dalhousie University, Halifax, NS, Canada) and performed using Illumina MiSeq 300 bp paired-end sequencing High-Throughput Sequencing (HTS) of the V6–V8 region with primers B969F and BA1406R (Comeau et al., 2017). Data were combined with those obtained from the 3-month fallow site studied previously (Verhoeven et al., 2016). Illumina reads were processed using the in-house SPONS-2 (Streamlined Processor Of Next-gen Sequences, version 2) pipeline with settings as previously described (Verhoeven and Dufour, 2017), with minor modifications. Briefly, the operational taxonomic unit (OTU) creation process using SWARM (Mahé et al., 2015) was modified to be more lenient [maximum differences between amplicons (*d*) increased from 1 to 3] to prevent an observed overestimation of found OTUs. In addition, alignment-based taxonomy assignment was replaced by the RDP naïve Bayesian classifier (Wang et al., 2007), retrained with the SILVA database release 132 (Quast et al., 2013), using a minimum bootstrap confidence estimate of 51% when assigning taxonomy. Raw reads are publicly available at the NCBI Sequence Read Archive under BioProject PRJNA503189.

### Bacterial Community Analysis

As recent studies have highlighted the deleterious effects of rarefying HTS data (McMurdie and Holmes, 2014) and the importance of treating HTS counts as compositional data (Gloor et al., 2017) we opted to analyze non-rarefied data using a compositional centered approach (Bian et al., 2017). Bacterial community richness was investigated by calculating the Hill’s series of diversity indices for each sample using Square-root transformed count data (Hill, 1973; Chao et al., 2014). Rather than providing a single diversity measurement, the recently rediscovered Hill numbers represent a continuum of possible diversity profiles, dictated by a set diversity order (*q*), which in turn determines the sensitivity of the Hill number to the relative frequencies of rare taxa (Ma, 2018; Ma and Li, 2018). Here, we calculated the Hill numbers (H, at *q* = 0, 1, and 2) which are analogous to the total number of species in a community (H_0_), the exponent of Shannon diversity (H_1_) and reciprocal of the Simpson’s index (H_2_). H_1_ and H_2_ can loosely be interpreted as the number of “typical” and “highly abundant” species, respectively. Differences in biodiversity indices across sample groups were assessed with multiple-comparison one-way ANOVAs using the Tukey multiple comparisons test; equal variance and data normality were confirmed using the Bartlett’s test and D’Agostino-Pearson normality test, respectively (GraphPad Prism v7). For exploratory beta-diversity analysis, low abundance OTUs were first filtered from each sample (minimum proportional abundance: 0.5%) and then zero counts were replaced with an imputed value using the *count zero multiplicative* method, using functions from the R packages CoDaSeq and zCompositions (Palarea-Albaladejo and Martín-Fernández, 2015), respectively. Zero count replaced data were then transformed with a *centered log ratio* (clr) transform using the CoDaSeq package (Gloor and Reid, 2016; Gloor et al., 2016), and subsequently used for singular value decomposition and PCA plot visualization, effectively plotting the inter-OTU variance between samples. Given the large variability in sample types present in the dataset, a preliminary exploration was performed by constructing a PCA plot from the clr-transformed count data, and subsequently, the optimal number of clusters (K) present in the distance matrix, as well as sample members of each cluster, were evaluated for *K* = 1 through *K* = 15 using the “total within sum of squares” approach. Hierarchical cluster analysis was performed on a Euclidean distance matrix calculated from the clr-transformed count data, using the “Ward.D2” agglomeration method. Differences between bacterial communities in clusters were tested using Permutational multivariate analysis of variance (PerMANOVA) with the “adonis” command in the vegan R package (Oksanen et al., 2018). For pairwise quantitative analyses (the detection of specific bacterial OTUs associated with each identified cluster, comparing specified pairs of clusters), differential abundance tests of clr-transformed posterior distributions of count data were used, generated by 128 Monte Carlo samples sourced from a Dirichlet distribution, performed with the ALDEx2 package in R (Fernandes et al., 2013, 2014). We considered that genera containing one or more OTUs with an effect size ≥1, and no detected effect sizes ≤1 with the contrasted group, were significantly associated with the tested cluster. We also examined which OTUs tended to co-occur in samples by calculating the ρ-metric from clr-transformed data as described above, using the *aldex2propr* function in the propr R package (Quinn et al., 2017). Associations between pairs of OTUs were considered significant where ρ > 0.60, and significant associations were subsequently visualized as networks of co-occurrence using Cytoscape (Shannon et al., 2003).

## Results

### Sequencing Results

High-Throughput Sequencing of the 16S rRNA gene was successful for 113 of 123 samples, yielding an average of 157,248 quality-check passed merged reads per sample (Supplementary Table S1). Sequence clustering with SWARM resulted in the creation of 24,267 OTUs. Rarefaction curve analysis of individual samples showed that a sufficient sequencing depth to accurately represent the microbial diversity was obtained for all samples, indicated by the plateauing of species richness rarefaction curves (Supplementary Figure S1). Of the complete set of OTUs, 371 were qualified as abundant, passing the minimal relative abundance filter of 0.5% (Supplementary Table S1).

### Bacterial Community Structure and Diversity

**FIGURE 1:**
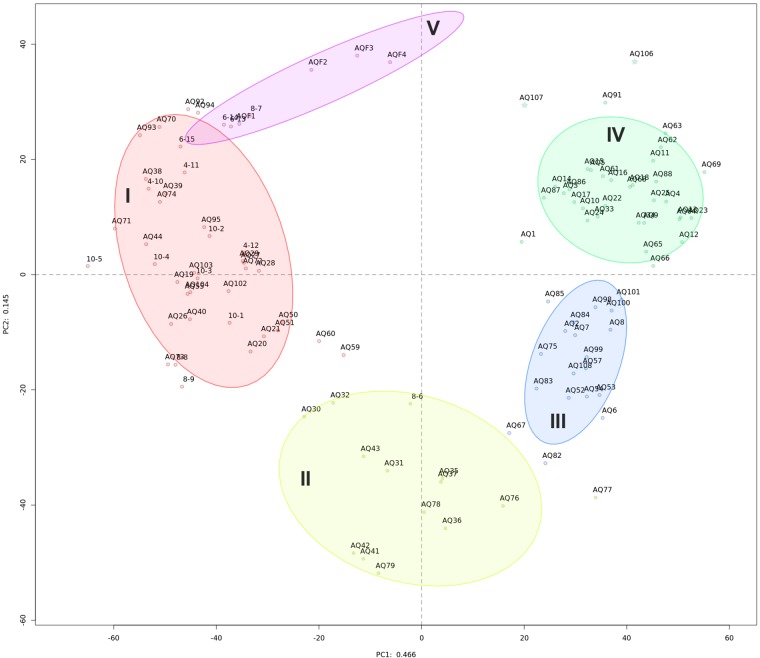
Compositional PCA plot of samples from this study. Dots, labeled with the sample identifier, are separated by distances signifying multivariate differences. Samples are colored by their cluster membership: high impact (I, red), recently disturbed (II, yellow), intermediate impact (III, blue), low impact (IV, green), and water (V, purple). Ellipses indicate a 75% confidence interval for each cluster. The variation explained by each axis is 46.6 and 14.5% for PC1 and PC2, respectively.

Initial exploration of bacterial community diversity using compositional PCA plot analysis did not reveal any correlation between sample groupings and available metadata (geographical location of site, production status, fallow or production length, or distance from cage, Supplementary Figure S2). Instead, five statistically distinct (*p* = 0.001 using perMANOVA) clusters were identified using unsupervised k-means clustering (Supplementary Figure S3), clearly delineated on the PCA plot (Figure 1 and Table 1).

**Table 1:** Unique clusters of samples recovered from aquaculture sites.

Cluster	1	2	3	4	5
**Interpretation**	High impact	Recently disturbed	Intermediate impact	Low impact	Water
**Cluster members (N)**	42	14	19	34	4
**Production samples (N)**	19	13	9	6	2
3 months	**7 (37%)**	**9 (69%)**	–	2 (33%)	2 (100%)
12 months	5 (26%)	4 (31%)	1 (11%)	**–**	–
24 months	**7 (37%)**		**8 (89%)**	**4 (67%)**	–
**Fallow samples (N)**	23	1	10	26	2
3 months	**14 (60%)**	**1 (100%)**	**–**	**–**	–
23 months	4 (18%)	**–**	4 (40%)	**18 (65%)**	2 (100%)
35 months	5 (22%)	**–**	**6 (60%)**	8 (30%)	–
**Distances from cage**					
0 m	**34 (81%)**	**9 (64%)**	5 (26%)	1 (3%)	2 (50%)
20 m	5 (12%)	3 (21%)	6 (32%)	5 (15%)	–
40 m	1 (2%)	2 (14%)	**7 (37%**)	5 (15%)	–
80 m	–	–	1 (5%)	**8** (**23%**)	–
120 m	2 (5%)	–	–	6 (18%)	–
160 m	–	–	–	6 (18%)	–
200 m	–	–	–	1 (3%)	(50%)
Far field	–	–	–	2 (6%)	–
**Mean %TOC**	28.96^∗^	16.44^∗^	7.84	4.41	ND
**Average H_0_**	746^∗^	1414^∗^	2172	2495	823
**Average H_1_**	423^∗^	933^∗^	1453^∗^	1860	542
**Average H_2_**	215^∗^	532^∗^	766^∗^	1171	305


*For each cluster, the number and percentage of constitutive samples from each production and fallow period (in months), as well as distance from cage (production and fallow sites combined) are reported. Bold typeface, mode; ND, not determined; H_*x*_, Hill biodiversity indices (H_*0*_, number of species; H_*1*_, exponent of Shannon diversity; H_*2*_, inverse Simpson); ^∗^significantly different from low cluster; %TOC = Percentage total organic carbon.*

**FIGURE 2:**
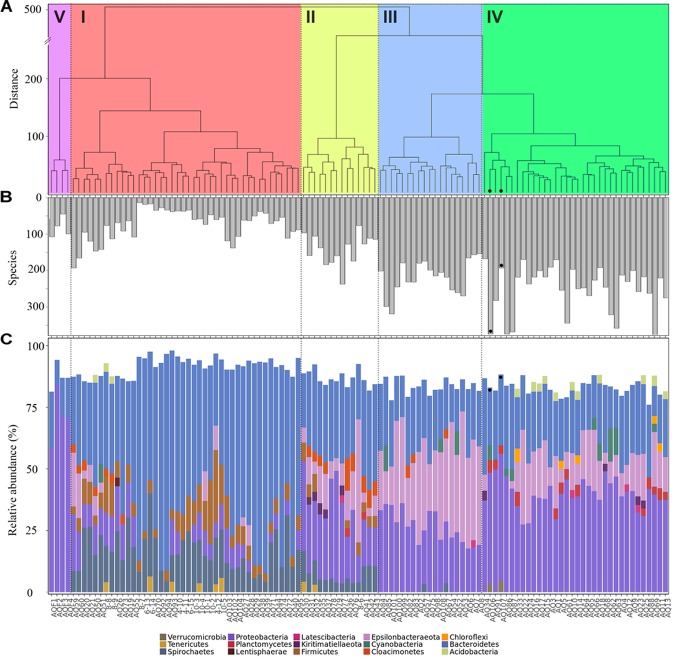
Properties of samples within aquaculture impact clusters. **(A)** Hierarchical clustering of a Euclidean distance matrix calculated from clr-transformed count data, agglomerated using “Ward.D2,” with clades are indicated as: high impact (I, red), recently disturbed (II, yellow), intermediate impact (III, blue), low impact (IV, green), and water (V, purple). **(B)** Biodiversity as number of species (H_0_) detected in each sample, and **(C)** phylum level community composition, shown as relative abundance per sample. Far-field samples are highlighted with a black dot in all panels.

Sample properties and biodiversity of cluster members were characterized to create a general classification (Table 1). The significant variance between the lowest and highest biodiversity estimates (clusters 1 and 4, H_0_: 746, H_1_: 423, H_2_: 215 versus 2495, 1860 and 1171, respectively, *p* < 0.001) was particularly evident. Based on the high biodiversity of cluster 4, and the results of hierarchical clustering of the Aitchison distance (Figure 2A), showing the far-field “reference” samples as members of cluster 4, we identified this cluster as “low impact” from aquaculture.

In contrast, cluster 1 was labeled as “high impact” based on the significantly lower biodiversity, the large proportion of samples that were sampled in close vicinity to cages (mode: 0 meters, 81% of members), in addition to the large Aitchison distance of this cluster to the other sample clusters, forming a separate clade (Figure 2A).

Clusters 2 and 3 were organized within the same overarching clade that included the low impact cluster (Figure 2A); however, all biodiversity measurements were still significantly lower when comparing cluster 2 to the low impact cluster (Table 1 and Figure 2B). This effect was similar for cluster 3; however, H_0_ (total species observed) was not significantly different. Furthermore, differences in production and fallow lengths between these clusters (mode of production length: 3 vs. 24 months; mode of fallow length: 3 vs. 35 months, for clusters 2 and 3, respectively) were observed (Table 1). Based on the H_0_ being similar to low impact values, the high mode of fallow length suggesting a more advanced recovery state, and low microbial community distance to the “low impact” cluster, we termed cluster 3 “intermediate impact,” indicative of samples resembling a “low impact” state. In contrast, as cluster 2 displayed a lower diversity measurement, in combination with the short production lengths, and low number of fallow samples, we termed this cluster “recently disturbed.”

Comparison of the phylum level taxonomic compositions (Figure 2C) showed large scale rearrangements of bacterial communities between clusters. Specifically, large increases in Firmicutes, Spirochaetes and Bacteroidetes, accompanied by a drastic decrease of Epsilonbacteraeota and Proteobacteria, were observed for samples within the high impact cluster. Similar changes, although less pronounced, were observed within the recently disturbed cluster. The intermediate impact cluster mostly mirrored the low impact cluster in terms of composition, with highly abundant phyla consisting of the Proteobacteria and Bacteroidetes. However, unlike the low impact cluster, intermediate impact samples had high relative abundances of the Epsilonbacteraeota. Interestingly, the two far-field samples collected fell into a small subclade of 6 samples within the low impact cluster, lacking abundant Epsilonbacteraeota as observed in other low impact samples.

The water samples, representing a different sample type, were highly dissimilar from other samples, both in clustering position (Figure 2A) as well as in phylum level taxonomic composition (Figure 2C).

### Correlation of Bacterial Clusters With Total Organic Carbon

**FIGURE 3:**
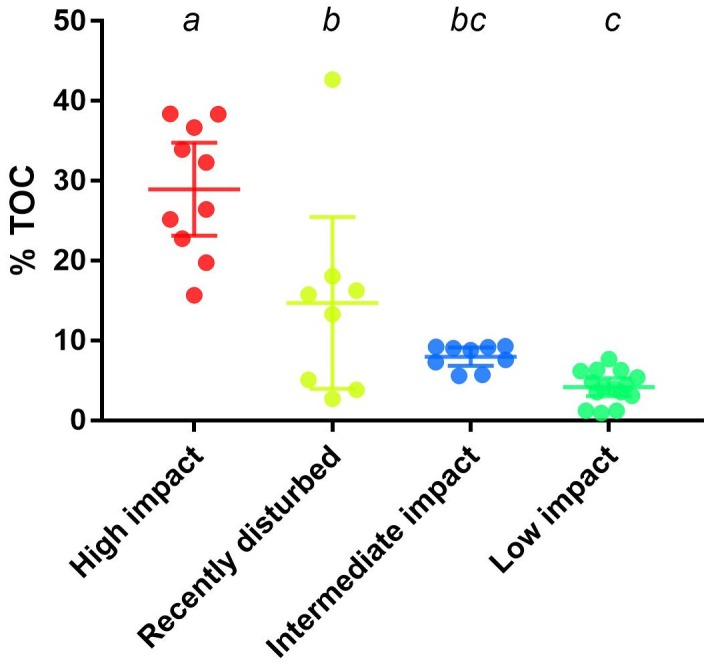
Percentage of total organic carbon across aquaculture impact clusters. Displayed are the measured percentages of total organic carbon (%TOC) from samples in bacterial derived distinct aquaculture impact clusters. Upper and lower whiskers indicate the 95% confidence interval while horizontal lines indicate the cluster %TOC mean. Letters above each cluster shows which groups are significantly (*p* < 0.05) different.

To strengthen the assigned classification of (bacterial community-based) impact clusters (Table 1), organic enrichment was investigated by comparing the %TOC across clusters. A significant difference (*p* < 0.0001, one-way ANOVA) was found in the mean %TOC values of impact clusters (Figure 3 and Supplementary Table S2). Multiple comparisons further revealed a significant difference between high impact (mean: 29% TOC) compared to recently disturbed (16.4% TOC, adjusted *p* = 0.0006), intermediate (7.84% TOC, adjusted *p* < 0.0001) and low impact (4.41% TOC, adjusted *p* < 0.0001) clusters. The %TOC of the recently disturbed cluster was also statistically different from low impact samples (adjusted *p* = 0.006), but not from intermediate impact (adjusted *p* = 0.20). Lastly, the mean %TOC of intermediate impact samples was not significantly different from low impact samples (adjusted *p* = 0.57).

### Biomarker Detection and Correlation

**FIGURE 4:**
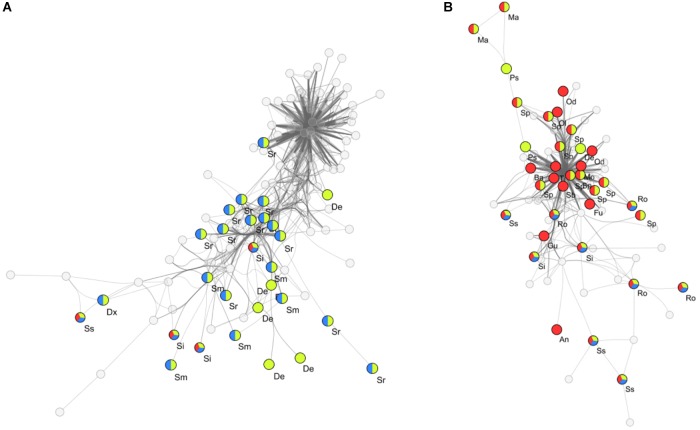
Co-occurring OTUs within flocculent matter samples. Two distinct co-occurrence clusters **(A,B)** between biomarkers of different aquaculture impact levels are seen. Empty circles represent non-biomarker bacterial OTUs while colored circles indicate biomarker OTUs and their associated impact groups (Red: high impact, Yellow: recently disturbed, Blue: intermediate impact; surface areas of circles are not indicative of a relative abundance), vertexes between OTUs represent a significant correlation (ρ > 0.65). Abbreviated genus names are also shown, Al, *Alloprevotella*; An, *Anaerophaga*; Ba, *Bacteroides*; Dx, *Desulfoconvexum*; De, *Desulforhopalus*; Fu, *Fusobacterium*; Gu, *Guggenheimella*; Ma, *Marinifilum*; Mo, *Mogibacterium*; Od, *Odoribacter*; Ol, *Oligosphaera*; Ps, *Psychromonas*; Ro, *Roseimarinus*; Sb, *Sedimentibacter*; Ss, *Sediminispirochaeta*; Sp, *Sphaerochaeta*; Si, *Spirochaeta*; Sm, *Sulfurimonas*; Sr, *Sulfurovum*; Tr, *Treponema*.

To determine key bacterial community members present within clusters I, II and III, a search for differentially abundant genera was conducted by performing pairwise comparisons between high impact, recently disturbed and intermediate impact clusters against the low impact cluster. With this approach we detected 28 genera which were differentially associated with one or more sample clusters (Table 2). Out of these genera, 12 were specific for high impact stations (including *Odoribacter, Alloprevotella, Sedimentibacter, Bacteroides*, and *Treponema*), 5 for recently disturbed stations (*Psychromonas, Desulfobacter, Desulforhopalus, Izimaplasma*, and *Fusibacter*), 7 associated with multiple clusters (including *Sphaerochaeta, Marinifilum*, and *Sulfurovum*) and 4 genera associated with all clusters other than low impact (*Roseimarinus, Desulfotalea, Sediminispirochaeta*, and *Spirochaeta*). Furthermore, correlation analysis using the ρ-metric revealed a strong correlation between biomarkers in two distinct co-occurrence webs (Figure 4). In web A, a total of 755 correlations between OTUs were found of which 140 (19%) were correlations between two biomarkers. Among biomarker to biomarker correlations, the most prominent connections were links between biomarkers associated with high impact (14%), recently disturbed (19%) as well as associations between biomarkers of high impact with recently disturbed markers (34%). Similarly, in web B 267 biomarker correlations were found within a total of 1377 (19%) correlations. The majority of these correlations occurred between OTUs associated with recently disturbed and intermediate impacts (82%).

**Table 2:** Biomarker genera associated with different aquaculture impact clusters.

Genus	High impact		Recently disturbed		Intermediate impact	
			
	M	A%	M	A%	M	A%
*Alloprevotella*	Y	4.28	N	<0.01	N	<0.01
*Anaerophaga*	Y	0.10	N	0.02	N	<0.01
*Bacteroides*	Y	1.34	N	0.39	N	0.01
*Desulfobacter*	N	0.84	Y	0.15	N	0.06
*Desulfobacterium*	N	0.26	Y	0.28	Y	0.23
*Desulfoconvexum*	N	0.19	Y	1.24	Y	0.84
*Desulfoplanes*	Y	0.12	N	0.02	N	<0.01
*Desulforhopalus*	N	0.69	Y	4.69	N	4.70
*Desulfotalea*	Y	0.39	Y	2.21	Y	0.22
*Draconibacterium*	N	0.19	Y	1.14	Y	0.82
*Fusibacter*	N	0.11	Y	0.53	N	0.09
*Fusobacterium*	Y	0.29	N	0.02	N	<0.01
*Guggenheimella*	Y	0.21	N	0.06	N	<0.01
*Izimaplasma*	N	0.20	Y	0.53	N	0.42
*Marinifilum*	Y	2.25	Y	1.77	N	0.60
*Mogibacterium*	Y	0.33	N	0.01	N	<0.01
*Odoribacter*	Y	16.99	N	0.08	N	0.02
*Oligosphaera*	Y	0.10	N	0	N	0
*Psychromonas*	N	2.35	Y	5.76	N	0.27
*Roseimarinus*	Y	2.05	Y	0.58	Y	0.09
*Sedimentibacter*	Y	1.42	N	0.11	N	<0.01
*Sediminispirochaeta*	Y	0.92	Y	1.57	Y	0.54
*Sphaerochaeta*	Y	10.28	Y	0.91	N	0.06
*Spirochaeta*	Y	0.91	Y	3.22	Y	1.11
*Sulfurimonas*	N	0.96	Y	3.63	Y	9.67
*Sulfurovum*	N	1.73	Y	7.74	Y	21.93
*Syntrophomonas*	Y	0.29	N	<0.01	N	0.01
*Treponema*	Y	1.33	N	<0.01	N	<0.01


*M: Marker status (indicates if given genus is a biomarker for respective group), Y: Yes, N: No, A%: Mean relative abundance of biomarker among members of cluster.*

### Effect of Fallowing on Biomarkers

**FIGURE 5:**
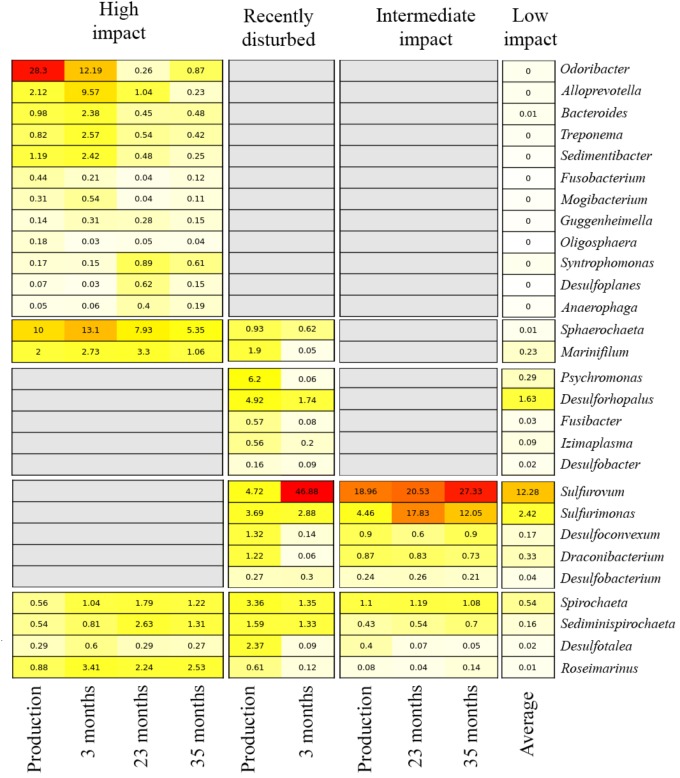
Relative abundance of biomarkers over variable fallowing lengths in aquaculture impact clusters. Each row indicates a biomarker genus. Numbers and color in cells show the average relative abundance (red: high, yellow: low, white: absent) per genus. Columns indicate the different fallow lengths at which the biomarker abundance was investigated, stratified by impact cluster; the average relative abundance of all production lengths is also shown. For the low impact cluster, the average abundance for all samples (i.e., all production and fallow periods combined) is presented for each bacterial genus. Gray boxes indicate that a genus is not considered a biomarker for a specific impact cluster.

The impact of the fallowing processes on benthic bacterial communities, and potential for fallow-based remediation, was investigated by tracking relative abundance changes of biomarkers over time within the high impact, recently disturbed and intermediate impact stations (Figure 5). At high impact stations, biomarker load was highest in production and short fallow length (3 months) samples, especially for abundant genera including the *Odoribacter* and *Sphaerochaeta*. While some of the impact biomarkers quickly diminished in abundance (*Odoribacter, Fusobacterium*, and *Oligosphaera*) after a short 3-month fallow period, others remained relatively constant or increased modestly throughout the 35-month fallowing period (including *Roseimarinus, Desulfotalea, Mogibacterium, Syntrophomonas, Guggenheimella*, and *Bacteroides*). For all biomarkers, the relative abundance remained above the mean level found in low impact sites during the entire range of fallow lengths (3–35 months). Within the intermediate impact cluster, several markers remained above low impact levels at the final time point (23 months, including *Spirochaeta, Sediminispirochaeta*, and *Desulfobacterium*). *Sulfurovum* and *Sulfurimonas* both increased in abundance after production and remained highly abundant during all periods of fallowing. We could not establish the effect of fallowing on the recently disturbed cluster, as it did not contain any long-term fallowing samples, fitting with the assessment that this represents a cluster currently in decline. Furthermore, the single fallow sample within this cluster displayed a unique divergent bacterial community composition (dominated by *Sulfurovum*).

### Detecting Flocculent Matter Biomarkers Within the Water Column

The usage of water samples as a proxy to detect flocculent matter bacterial signatures was investigated by comparing the relative abundance of previously identified flocculent matter biomarker taxa in each water sample. For water sampled at production site A, a clear increase in the relative abundance of several biomarkers associated with the “high impact” flocculent matter cluster was observed at 0 m from cage when compared to 200 m from cage. Most notable among the increased biomarker taxa where the *Psychromonas* (0 m: 6.84% vs. 200 m: 0.48%), *Odoribacter* (5.11% vs. 0.09%), *Bacteroides* (3.27% vs. 0.09%), *Sphaerochaeta* (2.23% vs. 0.07%), *Marinifilum* (0.89% vs. 0.05%), *Alloprevotella* (0.6% vs. 0.03%), *Sedimentibacter* (0.59% vs. 0%), and *Desulfotalea* (0.53% vs. 0.06%). Water samples from fallow site B did not show a high abundance of biomarkers from any impact cluster, and no evident shifts in biomarkers were seen comparing the distant and cage edge samples at this site.

## Discussion

In this study, we combined bacterial community analysis of natural sediment and flocculent matter underneath aquaculture operations in Newfoundland (Canada) to reveal benthic bacterial community changes over both a geographical scale and a temporal fallowing scale. Using unsupervised clustering of 16S rRNA bacterial community compositions, we revealed samples clustered in specific groups (Figure 1). Based on sample metadata as well as bacterial taxonomic composition and diversity, we suggest that these clusters of samples reflect the benthic bacterial community response to varying degrees of impact (high, intermediate, and low) that aquaculture operations exert on the benthic environment.

Mean %TOC, increases of which were used as proxy to assess aquaculture impact (Norði et al., 2011; Taranger et al., 2015), was significantly different between the bacterial community-based clusters. Most notably, a significant increase of TOC was observed in the high impact cluster compared to all other clusters, combined with significant declines in bacterial biodiversity alongside phylum level taxonomic re-arrangements such as large increases in Bacteroidetes, Firmicutes, Spirochaetes, and declines in Proteobacteria and Epsilonbacteraeota. Interestingly, the observed phylum level increases associated with presumed aquaculture impact are strikingly different from what has been reported in other studies, where Proteobacteria are often found in elevated abundances (Bissett et al., 2006; Dowle et al., 2015; Stoeck et al., 2018). However, bacterial responses are not necessarily universal between differing geographical regions (Dowle et al., 2015), and the absence of naturally occurring sediments (including an associated bacterial component) at our study sites may have driven the divergent phylum composition observed here.

Cluster membership of samples was not dictated by fallow length or production, but we observed that the average biodiversity and mode of cage distance increased when comparing high impact, intermediate impact and low impact clusters, respectively (Table 1). Correlations between distance from cage and benthic impact are not novel and have been observed in other bacterial – as well as macrobenthic – aquaculture surveillance studies (Keeley et al., 2014; Dowle et al., 2015; Hamoutene et al., 2015; Salvo et al., 2017). Surprisingly, the high impact cluster contained samples from a wide range of fallow and production lengths (3–35 months), indicating that benthic environments directly below pens remain highly impacted and do not return to a low or intermediate impact status, even after extensive fallowing, in line with what was previously reported for epibenthic fauna at the investigated sites (Salvo et al., 2017). While other studies have shown recovery within the first 3 months, and significant recovery after 2 years of fallowing (Keeley et al., 2014, 2015) such recovery was unfortunately not observed at the sites investigated here. This discrepancy might be due to differences in environmental conditions, such as current patterns, water temperatures or potentially the absence of a soft sediment-based resident bacterial community.

Interestingly, we also detected a small cluster composed mainly of stations where aquaculture operations had only recently started (3 months of production) after a fallow period. Although positioned within the same branch as “intermediate” and “low” impact clusters, a significantly lower biodiversity and higher %TOC was detected within these samples. As such, this branch could potentially indicate an actively changing benthic bacterial community, transitioning from a lower to a higher impact state while receiving organic enrichment from recently (re)started aquaculture operations.

Additional work is needed to strengthen the link between bacterial clusters and aquaculture impacts, such as additional environmental and chemical data. While the results showed in this study provide a promising proof of concept that community shifts can identify differential impact levels, including intermediate levels which is not currently achievable using visual surveys in our region (Salvo et al., 2017), further work is needed to fully verify the results presented here. Specifically, a higher resolution temporal scale could more accurately capture bacterial community fluctuations (including potential seasonal effects) and a more even sampling design could improve representation of “unique” samples such as those that are recently disturbed.

Comparing bacterial communities between the levels of impact, we were able to identify 28 biomarkers (differentially abundant genera) with affinity for one or more clusters (Table 2). Among the identified biomarkers were sulfate-reducing bacteria in the families Desulfobacteraceae and Desulfobulbaceae, often associated with organic matter enriched environments (Aylagas et al., 2017) and previously reported in the context of aquaculture impacts (Dowle et al., 2015). Other genera included the *Odoribacter, Alloprevotella* and *Syntrophomonas* associated with waste-water treatment plants, potentially indicating a fecal origin (McLellan et al., 2010; Aylagas et al., 2017). It is important to note that inferring functional importance of genera based on 16S rRNA data is speculative as bacteria with identical 16S rRNA gene sequences can have highly divergent genomes (Jaspers and Overmann, 2004); further studies are needed to establish the functional role of these biomarkers within flocculent matter. While the function of biomarkers cannot be ascertained, the co-occurrence of biomarkers (Figure 4) does suggest that aquaculture initiates complex environmental changes to which multiple genera respond, potentially in a co-dependant manner.

Tracking the presence and mean relative abundance of biomarkers shows that they do not all behave identically during the fallowing process. Several markers are short lived and decline to a low abundance after production or 3 months of fallowing (i.e., *Odoribacter, Alloprevotella, Bacteroides* in the high impact group), possibly indicating that these originate from fish feces or feed products and do not persist in the environment. In contrast, other genera (i.e., *Syntrophomonas, Sediminispirochaeta, Sulfurimonas*, and *Sulfurovum*) increase after production ceases. We previously hypothesized that flocculent matter breakdown might be a phased process, with different taxonomic groups taking precedence over time, and the shifts and co-occurrence patterns observed here support this theory (Verhoeven et al., 2016).

The differential abundance and cluster affinity of the identified biomarkers could make these specific genera potential candidates for biomonitoring, providing information on impact level and progression of fallowing. Water sampling – potentially following a purposeful disturbance of sediment/flocculent matter – could be an attractive alternative to grab sampling, especially at hard substrate-dominated sites. Due to the limited number of water samples in this study, no concrete conclusions can be drawn; however, the detection of high impact genera within the water samples is promising and suggests that a water sampling approach could be actionable.

In addition, the lifecycle of biomarkers suggests incomplete flocculent matter breakdown after 35 months of fallowing, as the majority of biomarkers remained significantly above low impact levels in all clusters. While this is not surprising, as other studies have shown full recovery can take up to 5 years or longer (Keeley et al., 2014), it is interesting to note that in this study some stations showed no significant recovery after approximately 3 years of fallowing, a timeframe which is commonly associated with substantial initial recovery (Keeley et al., 2015).

Our study demonstrates, along with other studies within this field (Bissett et al., 2006; Kawahara et al., 2009; Dowle et al., 2015; Aylagas et al., 2017; Cordier et al., 2018; Keeley et al., 2018; Stoeck et al., 2018), the immense potential for using bacteria as environmental indicators, allowing us to detect aquaculture impacts and track remediation over time. It also highlights the importance of recognizing that the local ecosystem can have a large influence on the initial composition and type of response of bacterial communities, as the results described herein differ significantly from those reported from aquaculture sites in other geographical locations (Kawahara et al., 2009; Dowle et al., 2015; Stoeck et al., 2018). Finally, we caution that coastal aquaculture on hard-bottom substrates might be especially prone to slow recovery, further underlining the importance of continued monitoring and the development of ecologically sustainable aquaculture practices.

## Author Contributions

JJV collected the samples, performed the data analysis, and authored the manuscript. FS coordinated the fieldwork, and conducted the sampling, meta-data collection, and analysis. RK collected the samples, processed the samples, and co-authored the manuscript. DH coordinated the study and fieldwork, and edited the manuscript. SD coordinated the study, assisted with data analysis, and co-authored the manuscript.

## Conflict of Interest Statement

The authors declare that the research was conducted in the absence of any commercial or financial relationships that could be construed as a potential conflict of interest.
