# Supplementary material for: Temporal Bacterial Surveillance of Salmon Aquaculture Sites Indicates a Long Lasting Benthic Impact With Minimal Recovery
Source: Front Microbiol. 2018 Dec 12;9:3054. doi: 10.3389/fmicb.2018.03054 (PMC6315143; doi:10.3389/fmicb.2018.03054)
Supplement: Supplementary file 1 [file Data_Sheet_1.pdf]

Temporal bacterial surveillance of salmon aquaculture sites indicates a long lasting benthic impact with minimal recovery

Joost T.P. Verhoeven[1,*], Flora Salvo[2], Robyn Knight[1], Dounia Hamoutene[2], Suzanne C. Dufour[1,*]

[1] Department of Biology, Memorial University of Newfoundland, 230 Elizabeth Ave., St. John's, NL, A1C 5S7, Canada.

[2] Fisheries and Oceans Canada, 80 East White Hills Rd, St. John's, NL, A1C 5X1, Canada

Contents

Supplementary figure S1: Rarefaction curve analysis of high-throughput sequencing data. 2

Supplementary figure S2: Compositional PCA plots of samples sequenced in this study. 3

Supplementary figure S3: Determination of optimal number of clusters within dataset 4

Supplementary Table S1: Samples sequenced in this study 5

Supplementary Table S2: Measurement of percentage total organic carbon within samples of distinct aquaculture impact clusters. 12

[*] Correspondence:

Mr. Joost T.P. Verhoeven

verhoevenjtp@gmail.com

Dr. Suzanne C. Dufour

sdufour@mun.ca

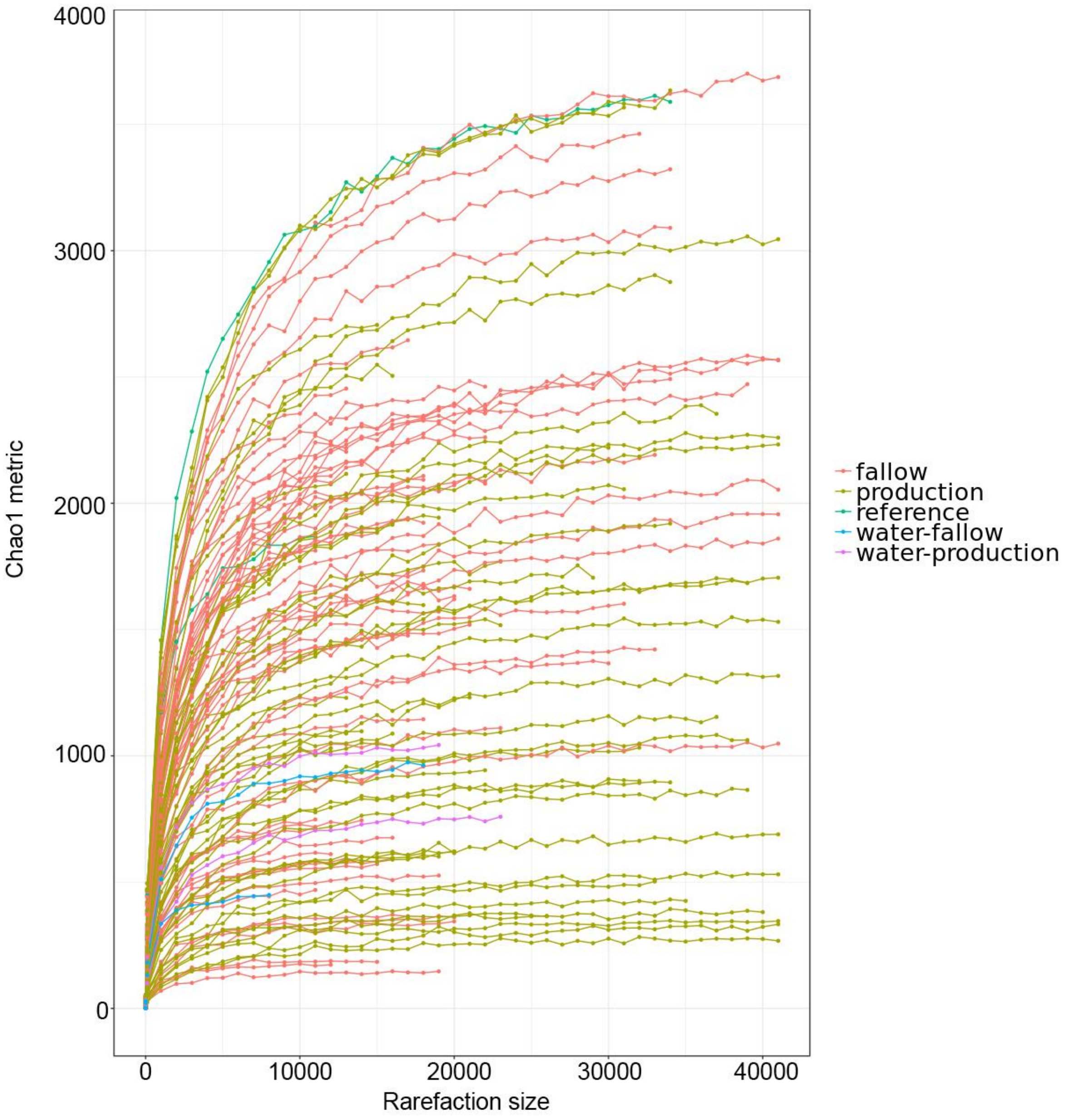

Supplementary figure S1: Rarefaction curve analysis of high-throughput sequencing data. Data points indicate mean alpha diversity at specified rarefaction sizes for the Chao1 metric. Color indicates sample type, red: fallow, orange: production, green: far-field samples, blue: fallow water, pink: production water.

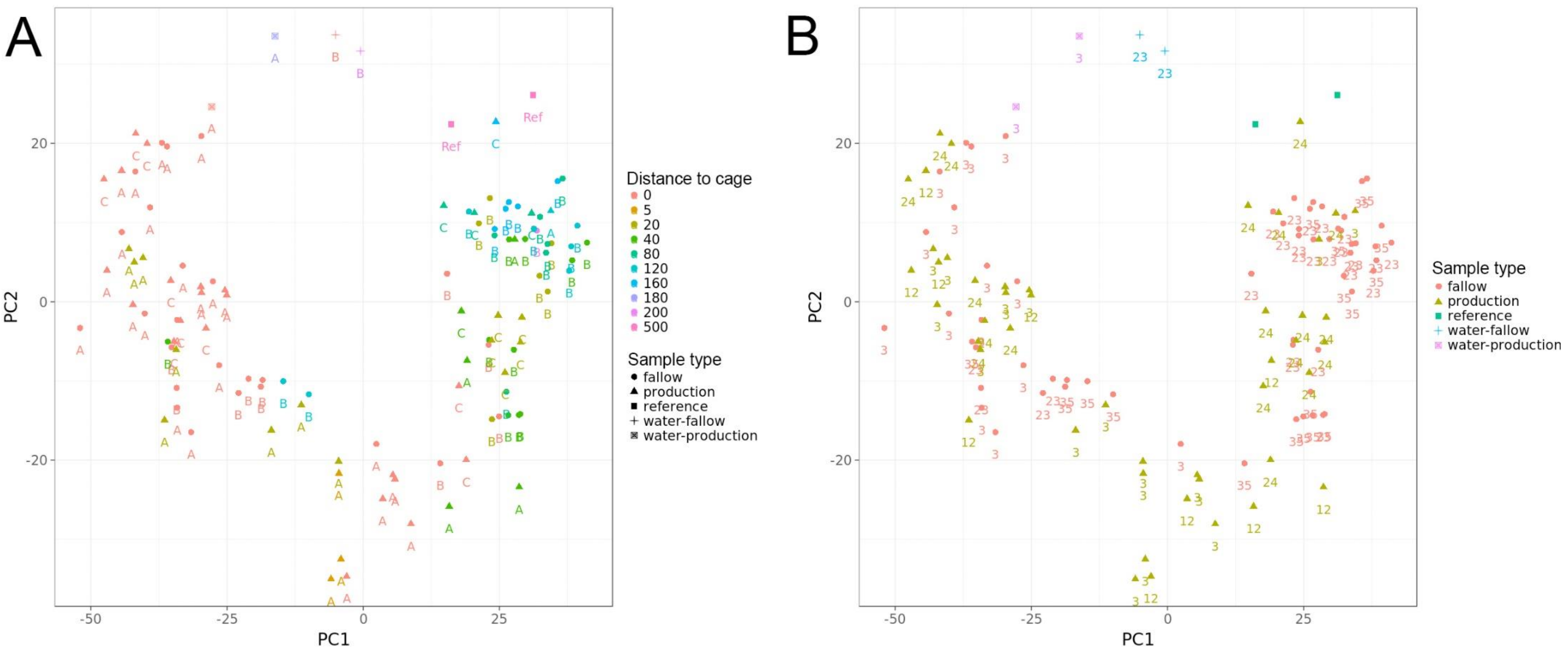

Supplementary figure S2: Compositional PCA plots of samples sequenced in this study. Symbols indicate samples and are separated by distances signifying multivariate differences. **(A)** Samples are color coded by distance to cage, with shape representing the sample type. Labels indicate the site from which a sample was collected. (**B)** Samples coded by type, with labels indicating either the fallow period in months (for fallow samples) or production period in months (for production samples).

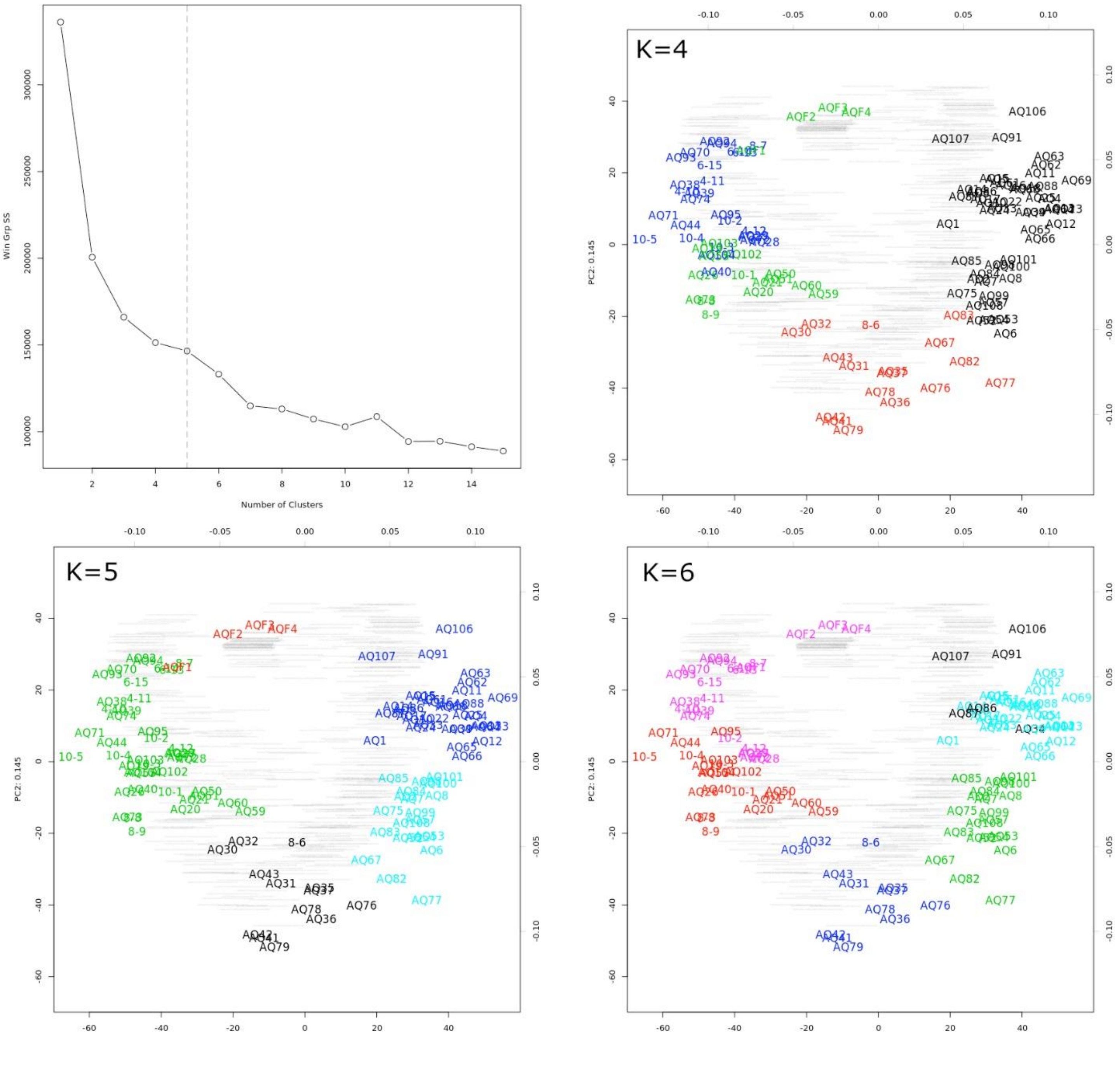

Supplementary figure S3: Determination of the optimal number of clusters within the dataset. Upper left graph shows the result of within cluster sums of squares analysis, indicating an optimal cluster number of K=5 using the elbow method. Additional graphs show compositional PCA plots of flocculent matter and water samples, colour-coded to represent k-means clustering where K=4, 5 and 6.

Verhoeven et. al. - Temporal bacterial surveillance of salmon aquaculture sites indicates a long lasting benthic impact with minimal recovery

Supplementary Table S1: Samples sequenced in this study

Sample	Site	Transect - Station	Type	Collection (YYYY-MM)	Cage distance	Fallow Length	Impact cluster	Production Length	Sequences	OTUs	OTUs (0.5%)
**10-1**	A	CE-1§	fallow	2015-08	0	3	I	NA	24756	737	217
**10-2**	A	CE-1§	fallow	2015-08	0	3	I	NA	22821	477	166
**10-3**	A	CE-1§	fallow	2015-08	0	3	I	NA	28346	602	197
**10-4**	A	CE-1§	fallow	2015-08	0	3	I	NA	29083	587	184
**10-5**	A	CE-1§	fallow	2015-08	0	3	I	NA	33366	541	160
**4-10**	A	CE-2§	fallow	2015-08	0	3	I	NA	33071	370	146
**4-11**	A	CE-2§	fallow	2015-08	0	3	I	NA	34165	379	133
**4-12**	A	CE-2§	fallow	2015-08	0	3	I	NA	34330	606	195
**6-13**	A	CE-3§	fallow	2015-08	0	3	I	NA	27243	195	66
**6-14**	A	CE-3§	fallow	2015-08	0	3	I	NA	24407	178	63
**6-15**	A	CE-3§	fallow	2015-08	0	3	I	NA	40626	346	124
**8-6**	A	CE-4§	fallow	2015-08	0	3	II	NA	22682	768	224
**8-7**	A	CE-4§	fallow	2015-08	0	3	I	NA	33468	147	54
**8-8**	A	CE-4§	fallow	2015-08	0	3	I	NA	26785	765	194
**8-9**	A	CE-4§	fallow	2015-08	0	3	I	NA	46809	1125	236
**AQ1**	B	3S-0m	fallow	2016-09	0	23	III	NA	18831	1695	200
**AQ10**	B	3S-80m	fallow	2016-09	80	23	III	NA	18366	1956	199
**AQ100**	C	T3-20m	production	2017-06	20	NA	IV	12	57554	2432	252
**AQ101**	C	T3-20m	production	2017-06	20	NA	IV	12	56732	3163	249
**AQ102**	C	T6-0m	production	2017-06	0	NA	I	12	75290	1374	263

**AQ103**	C	T6-0m	production	2017-06	0	NA	I	12	60694	1182	222
**AQ104**	C	T6-0m	production	2017-06	0	NA	I	12	54171	1061	231
**AQ105**	R	REF3	reference	2017-06	NA	NA	I	12	125	46	NA
**AQ106**	R	REF3	reference	2017-06	NA	NA	III	12	48784	3710	146
**AQ107**	R	REF3	reference	2017-06	NA	NA	III	12	14870	1921	104
**AQ108**	B	4W	fallow	2017-06	0	35	IV	NA	62859	2038	249
**AQ11**	B	3S-80m	fallow	2016-09	80	23	III	NA	16154	2191	177
**AQ12**	B	3S-120m	fallow	2016-09	120	23	III	NA	18366	2286	213
**AQ13**	B	3S-120m	fallow	2016-09	120	23	III	NA	25902	2730	203
**AQ14**	B	3S-120m	fallow	2016-09	120	23	III	NA	18363	2480	185
**AQ15**	B	3S-160m	fallow	2016-09	160	23	III	NA	29788	2162	178
**AQ16**	B	3S-160m	fallow	2016-09	160	23	III	NA	26065	2166	189
**AQ17**	B	3S-160m	fallow	2016-09	160	23	III	NA	23234	1991	200
**AQ18**	B	3S-200m	fallow	2016-09	200	23	III	NA	29887	1910	178
**AQ19**	B	8W-0m	fallow	2016-09	0	23	I	NA	19699	638	175
**AQ2**	B	3S-0m	fallow	2016-09	0	23	IV	NA	25914	1790	238
**AQ20**	B	8W-0m	fallow	2016-09	0	23	I	NA	24008	948	238
**AQ21**	B	8W-0m	fallow	2016-09	0	23	I	NA	31464	1190	250
**AQ22**	B	8W-40m	fallow	2016-09	40	23	III	NA	28860	1688	189

**AQ23**	B	8W-40m	fallow	2016-09	40	23	III	NA	28154	2558	224
**AQ24**	B	8W-40m	fallow	2016-09	40	23	III	NA	28670	2350	200
**AQ25**	B	8W-80m	fallow	2016-09	80	23	III	NA	22337	2156	225
**AQ26**	B	9N-0m	fallow	2016-09	0	23	I	NA	24769	689	174
**AQ27**	A	4S-0m	production	2016-10	0	NA	I	3	28398	621	184
**AQ28**	A	4S-0m	production	2016-10	0	NA	I	3	29032	633	199
**AQ29**	A	4S-0m	production	2016-10	0	NA	I	3	44264	507	175
**AQ3**	B	3S-20m	fallow	2016-09	20	23	III	NA	19372	1895	198
**AQ30**	A	4S-20m	production	2016-10	20	NA	II	3	27404	964	228
**AQ31**	A	4S-20m	production	2016-10	20	NA	II	3	32370	1581	257
**AQ32**	A	4S-20m	production	2016-10	20	NA	II	3	19223	1057	222
**AQ33**	A	4S-40m	production	2016-10	40	NA	III	3	18897	2169	193
**AQ34**	A	4S-120m	production	2016-10	120	NA	III	3	25642	1668	202
**AQ35**	A	4W-0m	production	2016-10	0	NA	II	3	21838	1370	227
**AQ36**	A	4W-0m	production	2016-10	0	NA	II	3	19698	1271	206
**AQ37**	A	4W-0m	production	2016-10	0	NA	II	3	36741	1828	244
**AQ38**	A	4W-20m	production	2016-10	20	NA	I	3	38992	385	115
**AQ39**	A	4W-20m	production	2016-10	20	NA	I	3	50298	434	130
**AQ4**	B	3S-20m	fallow	2016-09	20	23	III	NA	18631	2113	204

**AQ40**	A	4W-20m	production	2016-10	20	NA	I	3	50481	926	225
**AQ41**	A	1N-5m	production	2016-10	5	NA	II	3	31305	1265	210
**AQ42**	A	1N-5m	production	2016-10	5	NA	II	3	30184	1110	203
**AQ43**	A	1N-5m	production	2016-10	5	NA	II	3	25858	1140	223
**AQ44**	A	Center Cage	production	2016-10	0	NA	I	3	31973	639	189
**AQ49**	B	4W-0M	fallow	2017-06	0	35	I	NA	136	20	NA*
**AQ5**	B	3S-20m	fallow	2016-09	20	23	III	NA	19233	2523	190
**AQ50**	B	3S-0m	fallow	2017-06	0	35	I	NA	53297	1458	287
**AQ51**	B	3S-0m	fallow	2017-06	0	35	I	NA	50536	1414	280
**AQ52**	B	3S-20m	fallow	2017-06	20	35	IV	NA	58444	2587	262
**AQ53**	B	3S-40m	fallow	2017-06	40	35	IV	NA	73941	2675	264
**AQ54**	B	3S-40m	fallow	2017-06	40	35	IV	NA	63420	2514	267
**AQ55**	B	3S-40m	fallow	2017-06	40	35	I	NA	71478	1078	217
**AQ56**	B	3S-40m	fallow	2017-06	40	35	I	NA	280	48	NA*
**AQ57**	B	3S-80m	fallow	2017-06	80	35	IV	NA	32705	1733	247
**AQ58**	B	3S-80m	fallow	2017-06	80	35	IV	NA	108	18	NA*
**AQ59**	B	3S-120m	fallow	2017-06	120	35	I	NA	58048	1918	301
**AQ6**	B	3S-40m	fallow	2016-09	40	23	IV	NA	27466	1646	231

**AQ60**	B	3S-120m	fallow	2017-06	120	35	I	NA	50880	1647	286
**AQ61**	B	3S-160m	fallow	2017-06	160	35	III	NA	43836	3415	197
**AQ62**	B	3S-160m	fallow	2017-06	160	35	III	NA	46967	3186	207
**AQ63**	B	8W-80m	fallow	2017-06	80	35	III	NA	52420	3553	196
**AQ64**	B	8W-40m	fallow	2017-06	40	35	III	NA	39207	2457	205
**AQ65**	B	8W-20m	fallow	2017-06	20	35	III	NA	61463	2669	216
**AQ66**	B	8W-20m	fallow	2017-06	20	35	III	NA	49394	2248	215
**AQ67**	B	8W-0m	fallow	2017-06	0	35	IV	NA	61686	2169	268
**AQ68**	B	10S-120m	fallow	2017-06	120	35	III	NA	25024	2440	206
**AQ69**	B	10S-120m	fallow	2017-06	120	35	III	NA	64042	3796	185
**AQ7**	B	3S-40m	fallow	2016-09	40	23	IV	NA	28917	1558	197
**AQ70**	A	1N-0m	production	2017-06	0	NA	I	12	55910	354	123
**AQ71**	A	1N-0m	production	2017-06	0	NA	I	12	82688	712	168
**AQ72**	A	1N-0m	production	2017-06	0	NA	I	12	54332	1109	251
**AQ73**	A	1N-20m	production	2017-06	20	NA	I	12	41229	915	204
**AQ74**	A	1N-20m	production	2017-06	20	NA	I	12	65870	543	162
**AQ75**	A	1N-40m	production	2017-06	40	NA	IV	12	48032	1975	202
**AQ76**	A	1N-40m	production	2017-06	40	NA	II	12	59619	1730	219

**AQ77**	A	4S-40m	production	2017-06	40	NA	II	12	73291	2357	216
**AQ78**	A	Center Cage	production	2017-06	0	NA	II	12	74317	1767	220
**AQ79**	A	Center Cage	production	2017-06	0	NA	II	12	74228	1588	205
**AQ8**	B	3S-40m	fallow	2016-09	40	23	IV	NA	23460	1530	207
**AQ80**	A	4W-0m	production	2017-06	0	NA	IV	12	222	65	NA*
**AQ81**	C	T4-0m	production	2017-06	0	NA	IV	12	200	42	NA*
**AQ82**	C	T4-0m	production	2017-06	0	NA	IV	12	58235	2297	251
**AQ83**	C	T4-0m	production	2017-06	0	NA	IV	12	41782	2301	266
**AQ84**	C	T4-20m	production	2017-06	20	NA	IV	12	29228	2001	231
**AQ85**	C	T4-40m	production	2017-06	40	NA	IV	12	56928	2964	283
**AQ86**	C	T4-80m	production	2017-06	80	NA	III	12	53662	3711	249
**AQ87**	C	T4-80m	production	2017-06	80	NA	III	12	53781	3654	274
**AQ88**	C	T4-80m	production	2017-06	80	NA	III	12	21440	2605	190
**AQ89**	C	T4-120m	production	2017-06	120	NA	III	12	89	20	NA*
**AQ9**	B	3S-80m	fallow	2016-09	80	23	III	NA	24621	1989	208
**AQ90**	C	T4-120m	production	2017-06	120	NA	III	12	95	18	NA*
**AQ91**	C	T4-160m	production	2017-06	160	NA	III	12	21921	2799	165
**AQ92**	C	T2-M1	production	2017-06	0	NA	I	12	64267	283	79

**AQ93**	C	T2-M1	production	2017-06	0	NA	I	12	56187	389	131
**AQ94**	C	T2-M1	production	2017-06	0	NA	I	12	91118	345	107
**AQ95**	C	T2-M1	production	2017-06	0	NA	I	12	62069	882	220
**AQ96**	C	T3-0m	production	2017-06	0	NA	I	12	105	28	NA*
**AQ97**	C	T3-0m	production	2017-06	0	NA	I	12	167	41	NA*
**AQ98**	C	T3-20m	production	2017-06	20	NA	IV	12	40424	2133	233
**AQ99**	C	T3-20m	production	2017-06	20	NA	IV	12	39551	1780	245
**AQF1**	A	4S-0m	water production	2016-10	0	NA	V	3	23730	1071	225
**AQF2**	A	4S-180m	water production	2016-10	180	NA	V	3	32783	776	136
**AQF3**	B	3S-0m	water fallow	2016-09	0	23	V	NA	9906	455	96
**AQF4**	B	3S-200m	water fallow	2016-09	200	23	V	NA	22900	992	168

CE: Cage edge**, R**: Far-field "reference" site, **NA**: Not available/applicable, *****: Sample excluded due to low read count, §: sample previously described in Verhoeven et al (2015).

Supplementary Table S2: Measurement of percentage total organic carbon within samples of distinct aquaculture impact clusters.

Site	Transect - Station	Average %TOC	Standard deviation	N samples	Impact group
B	8W-0m	22.79	1.12	5	High
A	Center cage	19.78	3.62	3	High
A	4S-0m	26.44	2.81	2	High
A	4W-20m	38.40	1.08	2	High
B	3S-0m	15.67	0.22	2	High
B	3S-40m	32.33	1.51	2	High
B	3S-120m	25.20	0.78	2	High
A	1N-0m	33.94	1.09	2	High
A	1N-0m	38.34	0.14	2	High
A	1N-20m	36.69	1.08	2	High
B	3S-0m	5.61	0.19	2	Intermediate
B	3S-40m	5.73	0.15	2	Intermediate
B	4W CE	9.19	0.66	3	Intermediate
B	4W CE	7.58	0.86	2	Intermediate
B	3S-20m	9.05	0.35	2	Intermediate
B	3S-40m	9.32	0.57	2	Intermediate
B	3S-80m	7.33	0.32	2	Intermediate
B	8W-0m	9.22	0.45	2	Intermediate
A	1N-40m	8.83	0.44	2	Intermediate
B	3S-0m	7.69	0.83	4	Low
B	3S-20 m	4.45	0.97	2	Low
B	3S-80m	5.38	0.58	4	Low
B	3S-120	4.79	1.70	2	Low
B	3S-160	3.57	0.18	2	Low
B	8W-40m	4.16	NA	1	Low
B	8W-80	3.58	0.27	2	Low
REF	REF	0.93	0.11	2	Low
B	3S-160m	3.11	0.07	2	Low
B	8W-80m	3.96	0.21	2	Low
B	8W-40m	6.37	0.84	2	Low
B	8W-20m	6.33	1.02	2	Low
B	10S-120m	6.18	0.86	2	Low

REF	REF	1.23	0.17	2	Low
A	4S-20m	15.78	1.21	5	Recently disturbed
A	1N-5m	13.32	2.13	3	Recently disturbed
A	4W-0m	42.69	0.17	2	Recently disturbed
A	Center cage	18.05	2.22	2	Recently disturbed
A	Center cage	16.29	0.62	2	Recently disturbed
A	4W-0m	2.72	0.14	2	Recently disturbed
A	1N-40m	5.11	0.73	2	Recently disturbed
A	4S-40m	3.86	0.40	2	Recently disturbed

%TOC: Percentage total organic carbon, **REF**: Samples collected from far-field “reference” stations
